# Identifying Distinct Trajectories and Predictors of Professional Identity in Nursing Interns: A Prospective Cohort Study

**DOI:** 10.1002/nop2.70738

**Published:** 2026-08-12

**Authors:** Zhengju Chen, Peng Shao, Hongrang Chen, Biaoxin Zhang

**Affiliations:** ^1^ Department of Pediatrics The First Affiliated Hospital of Anhui Medical University Hefei Anhui China; ^2^ Department of Hepatobiliary, Pancreatic and Transplant Surgery II The First Affiliated Hospital of Anhui Medical University Hefei Anhui China; ^3^ Nursing Department The First Affiliated Hospital of Anhui Medical University Hefei Anhui China

**Keywords:** latent class, nursing interns, professional identity, prospective study, Trajector

## Abstract

**Aim:**

Clinical internships represent a critical phase for shaping professional identity in undergraduate nursing students, which significantly influences their career intentions, workforce retention, and the stability of the nursing profession. However, limited research has examined the dynamic, heterogeneous developmental patterns of professional identity throughout the internship period. This study aimed to identify distinct trajectory classes of professional identity and their predictors, with the goal of informing targeted interventions to strengthen professional identity and reduce workforce attrition.

**Design:**

Prospective cohort study.

**Methods:**

This prospective cohort study enrolled undergraduate nursing interns from two tertiary hospitals in Anhui Province, China, from January to December 2024. Professional identity was measured using the Professional Identity Questionnaire for Nurse Students (PIQNS) at three time points: the end of the 3rd (early), 6th (middle), and 9th (late) months of internship. We used Latent Class Growth Modelling (LCGM) to identify distinct developmental trajectories. Univariate analyses and binary logistic regression were performed to examine factors associated with trajectory membership.

**Results:**

Of the 251 interns who completed all assessments, LCGM identified two distinct trajectory classes: a low‐level group (*n* = 128, 51%), characterized by low initial scores, a mild mid‐internship rise, and a subsequent moderate decline; and a high‐level group (*n* = 123, 49%), characterized by consistently high scores, a slight mid‐internship increase, and only minor late‐stage fluctuation. The logistic regression results revealed that voluntarily choosing nursing as a university major (reference = no, OR = 0.195, *p* = 0.002), liking nursing (reference = dislike, OR = 0.260, *p* < 0.001), and having leadership experience (reference = no, OR = 0.446, *p* = 0.007) were all associated with lower odds of being classified into the low‐level group (all OR < 1).

**Conclusion:**

The development of professional identity among nursing interns exhibits significant inter‐individual heterogeneity. Clinical educators and academic instructors should consider these distinct trajectory classes when designing personalized, stage‐specific interventions to enhance professional identity and mitigate workforce attrition in nursing.

**Implications for the Profession:**

These findings provide actionable guidance for clinical preceptors and academic instructors to identify interns at risk of professional identity decline, especially those with non‐voluntary major choice, low professional interest, or no leadership experience. Educators should implement stage‐specific support: pre‐internship orientation to manage expectations, mid‐internship competency feedback and peer mentoring, and late‐internship career counselling and resilience training to reduce burnout and employment pressure. Such targeted interventions may enhance professional identity stability and improve nursing workforce retention.

**Patient or Public Contribution:**

No patient or public contribution.

## 1 Introduction

Clinical internship is a mandatory and critical phase in undergraduate nursing education, serving as the primary pathway for students to transition from academic settings to professional practice (Khattar et al. 2025). In China, this typically entails a 10–12 month full‐time clinical placement in a teaching hospital, during which students rotate through major departments (e.g., internal medicine, surgery, emergency, ICU) under preceptor supervision to develop clinical skills, critical thinking, and professional values (Rodríguez‐García et al. 2021). This period is characterized by dual transitions in both role and environment, and failure to adapt can negatively impact students' career intentions and the stability of the nursing workforce (Zeng et al. 2022).

Professional identity refers to an individual's sense of their professional attributes, beliefs, values, and motivations. Research demonstrates that professional identity significantly influences nursing interns' mental health, work attitudes, career retention intentions, and quality of patient care (Wu et al. 2020). The internship period is a pivotal juncture in nursing interns' professional identity formation. Undergraduate nursing interns, who often hold higher professional expectations due to their advanced education (Wu et al. 2020), frequently encounter a gap between these expectations and the realities of clinical practice. This discrepancy, as documented by Najafi Kalyani et al. (Najafi Kalyani et al. 2019), may manifest as fear, confusion, or self‐doubt, ultimately eroding professional identity. If left unaddressed, these negative experiences may weaken interns' commitment to nursing practice and increase their risk of leaving the profession after graduation (Zeng et al. 2022), highlighting the need for targeted support during this phase. Although numerous studies have examined nursing interns' professional identity, most have employed cross‐sectional designs (Zeng et al. 2023; Mukherjee et al. 2024; Zhao, Liang, et al. 2024), leaving the dynamic evolution of this construct throughout the internship largely unexplored. Understanding these temporal dynamics is essential for developing stage‐specific interventions. Furthermore, the potential heterogeneity in developmental trajectories has not been systematically investigated.

The Latent Class Growth Model (LCGM) is a common statistical approach for longitudinal trajectory analysis. This model is adept at identifying potential categories representing distinct trajectories (Zoccali and Tripepi 2025; Zhao, Wang, et al. 2024). To address this gap, this study employed a LCGM to: (1) identify distinct subgroups of undergraduate nursing interns based on their professional identity trajectories over a 9‐month internship, and (2) examine predictors of subgroup membership. The findings will provide a nuanced evidence base for developing targeted, stage‐specific interventions to bolster professional identity and mitigate talent attrition.

### 1.1 Research Questions

What are the distinct trajectories of professional identity development among undergraduate nursing interns over a 9‐month internship?

Which factors (e.g., voluntary major choice, class cadre experience) predict membership in these trajectory subgroups?

### 1.2 Research Purpose

The findings will provide a nuanced evidence base for developing targeted, stage‐specific interventions by academic institutions and hospitals to bolster interns' professional identity and mitigate talent attrition in the nursing profession.

## 2 Methods

### 2.1 Research Design

This non‐experimental prospective cohort study employed convenience sampling. Participants were undergraduate nursing interns who commenced their internship between January and December 2024 at two tertiary hospitals in Anhui Province, China.

Inclusion criteria for participants included: (1) full‐time undergraduate nursing interns admitted through the National College Entrance Examination (Gaokao); (2) signed written informed consent and voluntary participation in the study; (3) currently in the clinical internship stage (no prior clinical work or internship experience).

Exclusion criteria included: (1) withdrawal from the study for any reason (e.g., personal leave, transfer); (2) poor quality of questionnaire completion (e.g., regularized responses, completion time < 1 min); (3) absence from internship posts for more than 1 month due to illness or personal reasons.

Sample size was estimated using the formula for longitudinal studies, assuming a standard deviation (SD) of 15, a two‐sided significance level of 0.05, and a 20% attrition rate. This calculation yielded a minimum requirement of 247 participants. All participants were undergraduate nursing interns who met the study's inclusion criteria, and written informed consent was obtained from each individual before their enrolment.

### 2.2 Survey Tools

Two tools were used for data collection: a self‐designed General Information Questionnaire and the Professional Identity Questionnaire for Nurse Students (PIQNS).

#### 2.2.1 General Information Questionnaire

Based on previous research (Rodríguez‐García et al. 2021; Zeng et al. 2022, 2023; Wu et al. 2020; Najafi Kalyani et al. 2019; Mukherjee et al. 2024; Zhao, Liang, et al. 2024), we developed a structured questionnaire to collect data on potential influencing factors, including: (1) demographic characteristics (gender, age, hometown [urban/rural]); (2) educational background (academic stream [science/humanities], whether nursing was their first‐choice major); (3) career‐related attitudes and support (attitude toward the nursing major [‘Like,’ ‘General,’ ‘Dislike’], family support for a nursing career [Yes/No]); and (4) other factors (leadership experience [class cadre or intern group leader, Yes/No], family members in healthcare [Yes/No], prior hospitalization or caregiving experience [Yes/No]).

#### 2.2.2 Professional Identity Questionnaire for Nurse Students (PIQNS)

Professional identity was measured using the PIQNS, developed by (Hao 2011). This questionnaire assesses nursing interns' professional identity across five dimensions: professional self‐concept, retention benefits and turnover risks, social support and self‐reflection, autonomy in career choice, and social persuasion. It consists of 17 items, one reverse‐coded item (Item 12), and uses a 5‐point Likert scale (1 = ‘strongly disagree’ to 5 = ‘strongly agree’). Total scores range from 17 to 85, with higher scores indicating stronger professional identity.

#### 2.2.3 Reliability and Validity

The PIQNS has demonstrated satisfactory internal consistency (Cronbach's *α* = 0.827) and construct validity (CFI = 0.91, RMSEA = 0.068) in its original validation study (Hao 2011). In the current sample, the scale also showed acceptable reliability, with a Cronbach's *α* of 0.812.

### 2.3 Data Collection

Nursing interns who met the inclusion and exclusion criteria were invited to participate. Prior to data collection, all participants were informed about the study's purpose, procedures, their right to withdraw at any time without any penalty to their academic or internship evaluation, and the measures in place to ensure confidentiality. Written informed consent was obtained from all participants.

Two research assistants administered the surveys at three time points: the early stage (T1, at the end of the 3rd month), middle stage (T2, at the end of the 6th month), and late stage (T3, at the end of the 9th month). At T1, paper‐based questionnaires were distributed on‐site and collected immediately after completion. For T2 and T3, to facilitate follow‐up and reduce participant burden, a combination of online (via the Wenjuanxing platform, a Chinese online survey tool) and paper‐based questionnaires was used. To acknowledge participants' time, a small gift (valued at approximately RMB 20) was offered upon completion of each survey. Participation was entirely voluntary, and incentives were provided solely for completion, irrespective of response content, to minimise perceived coercion.

### 2.4 Data Analysis

Data were initially screened using Excel 2021. Questionnaires with patterned responses (e.g., identical answers across all items), a completion time of less than 1 min, or more than 20% missing data were excluded. All retained data were double‐entered and cross‐checked to minimise input errors. Statistical analyses were performed using SPSS 27.0 and Mplus 8.3, with a two‐tailed significance level of *α* = 0.05.

### 2.5 Descriptive Statistics

Normality of continuous variables was assessed using the Shapiro–Wilk test. Normally distributed data are presented as mean ± SD, while non‐normally distributed data are presented as median (interquartile range). Categorical variables are summarised as frequencies and percentages. Between‐group differences were compared using the chi‐square test for categorical variables and the independent‐samples *t*‐test for normally distributed continuous variables.

LCGM was employed to identify latent subgroups (classes) with distinct developmental trajectories of professional identity scores across the three time points (Zoccali and Tripepi 2025; Zhao, Wang, et al. 2024). Three equally spaced measurement time points were labelled T1 (baseline), T2, and T3, and time scores were coded as 0, 1, 2. Only a linear growth specification was adopted, as quadratic terms cannot be estimated with only three repeated measurements. Following classic LCGM assumptions, within‐class variances of intercept and slope were fixed to zero, and residual variances were freely estimated across latent classes. Models with 1 to 5 classes were sequentially fitted. Model fit was evaluated using the Akaike information criterion (AIC), Bayesian Information Criterion (BIC), sample‐size adjusted BIC (aBIC), entropy, and the average posterior probability for each class. Entropy values > 0.80 and average posterior probabilities > 0.70 were considered indicative of satisfactory classification accuracy. Given the limitation of only three measurement waves, the model cannot capture potential nonlinear inflection points. Trajectories reflect linear interpolation between observed time points; therefore, cautious interpretation is warranted.

Binary logistic regression was performed to identify predictors of trajectory group membership. Variables with *p* < 0.05 in univariate analyses (chi‐square test or *t*‐test) were included as independent variables in the regression model. The outcome was coded as 1 for the low‐level group and 0 for the high‐level group; thus, OR < 1 indicates lower odds of belonging to the low‐level group.

## 3 Results

### 3.1 Characteristics of Study Participants

Of the 275 undergraduate nursing interns initially recruited, 24 withdrew (attrition rate: 8.7%), yielding a final sample of 251 interns who completed all three assessments (response rate: 91.3%). The sample included 30 males (12.0%) and 221 females (88.0%). The average age of participants was 21.73 ± 1.40 years. Other basic information is detailed in Table 1.

**TABLE 1:** Characteristics of the study sample and comparison between the two trajectory groups (*N* = 251).

Characteristic	Total (*N* = 251)	Low‐level group (*N* = 128)	High‐level group (*N* = 123)	*t*/*χ*2	*P*
Age, (mean ± SD)	21.73 ± 1.40	21.55 ± 1.27	21.92 ± 1.50	2.079	0.039
Sex, *n* (%)				0.256	0.613
Male	30 (12.0%)	14 (10.9%)	16 (13.0%)		
Female	221 (88.0%)	114 (89.1%)	107 (87.0%)		
Hometown, *n* (%)				0.037	0.847
City	124 (49.4%)	64 (50.0%)	60 (48.8%)		
Village	127 (50.6%)	64 (50.0%)	63 (51.2%)		
Disciplinary Background, *n* (%)				0.101	0.750
Science	82 (32.7%)	43 (33.6%)	39 (31.7%)		
Art	169 (67.3%)	85 (66.4%)	84 (68.3%)		
Nursing as first‐choice major, *n* (%)				30.915	< 0.001
Yes	198 (78.9%)	83 (64.8%)	115 (93.5%)		
No	53 (21.1%)	45 (35.2%)	8 (6.5%)		
Attitude toward nursing major, *n* (%)				40.986	< 0.001
Like	110 (43.8%)	31 (24.2%)	79 (64.2%)		
General	129 (51.4%)	88 (68.8%)	41 (33.3%)		
Dislike	12 (4.8%)	9 (7.0%)	3 (2.4%)		
Intention to pursue nursing career, *n* (%)				5.321	0.021
Yes	228 (90.8%)	111 (86.7%)	117 (95.1%)		
No	23 (9.2%)	17 (13.3%)	6 (4.9%)		
Leadership experience (class cadre/intern leader), *n* (%)				6.690	0.010
Yes	71 (28.3%)	33 (25.8%)	38 (30.9%)		
No	180 (71.7%)	95 (74.2%)	85 (69.1%)		
Family members in healthcare, *n* (%)				0.853	0.356
Yes	115 (45.8%)	55 (43.0%)	60 (48.8%)		
No	136 (54.2%)	73 (57.0%)	63 (51.2%)		
Prior hospitalization/caregiving experience, *n* (%)				1.058	0.304
Yes	171 (68.1%)	91 (71.1%)	80 (65.0%)		
No	80 (31.9%)	37 (28.9%)	43 (35.0%)		
Family support, *n* (%)				1.568	0.211
Yes	236 (94.0%)	118 (92.2%)	118 (95.9%)		
No	15 (6.0%)	10 (7.8%)	5 (4.1%)		

*Note:* ‘Attitude toward nursing major’ was assessed by the question: ‘Do you like the nursing major?’ with responses of Like, General, or Dislike. ‘Family support’ was assessed by the question: ‘Do your family members support your decision to work as a nurse?’ (Yes/No).

### 3.2 The Trajectory of Professional Identity in Nursing Interns

The mean professional identity scores at T1, T2, and T3 were 59.45 ± 11.66, 66.04 ± 12.13, and 63.39 ± 13.02, respectively. We fitted LCGM models with one to four classes to determine the optimal trajectory solution. Model fit indices are presented in Table 2. As shown in Table 2, the AIC, BIC, and aBIC decreased with increasing class numbers. The LMR test was significant for all models. However, in the 3‐ and 4‐class solutions, at least one class comprised less than 5% of the sample, indicating over‐extraction. Considering parsimony, classification accuracy (entropy = 0.806), and interpretability, the 2‐class solution was selected as the optimal model. The average posterior probabilities for the two classes were 0.89 and 0.90, respectively, suggesting excellent class separation.

**TABLE 2:** Fitting indices of the LCGM model for professional identity of nursing interns.

Model	AIC	BIC	aBIC	entropy	LMR(*P*)	BLRT(*P*)	Class proportions
1	5941.610	5959.237	5943.387				
2	5630.532	5658.736	5633.375	0.806	0.024	< 0.001	0.49/0.51
3	5476.512	5515.292	5480.421	0.891	0.039	< 0.001	0.598/0.386/0.016
4	5252.900	5302.257	5257.875	0.931	< 0.001	< 0.001	0.016/0.378/0.203/0.403

*Note:* AIC (Akaike Information Criterion) and BIC (Bayesian Information Criterion) are model fit indices; smaller values indicate better model fit. entropy > 0.8 indicates good classification accuracy. *p* < 0.05 indicates the k‐class model is superior to the k‐1 class model.

Abbreviations: aBIC, adjusted BIC; BLRT, Bootstrap Likelihood Ratio Test; LMR, Lo–Mendell–Rubin likelihood ratio test.

**FIGURE 1:**
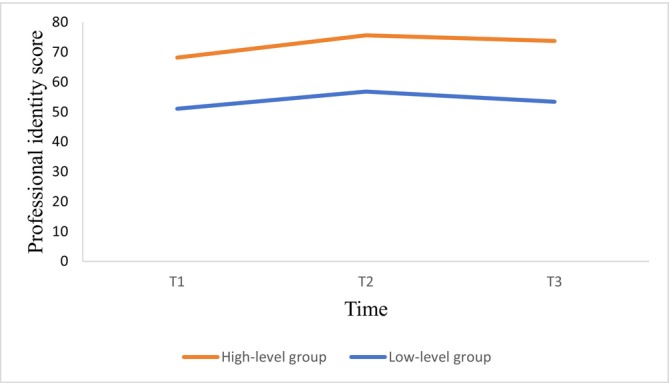
Two distinct trajectories of professional identity scores among nursing interns over the 9‐month internship period.

As illustrated in Figure 1, the two trajectory classes were characterized as follows: Class 1 (Low‐level group, *n* = 128, 51%): This class started with low professional identity scores (51.07 ± 7.64), showed a moderate increase in the middle stage (56.81 ± 8.38), and then experienced a sharp decline in the late stage (53.41 ± 8.26).

Class 2 (High‐level group, *n* = 123, 49%): This class maintained consistently higher scores throughout the internship, with a slight increase at T2 (from 68.18 ± 8.21 to 75.64 ± 6.79) and a minor decrease at T3 (73.76 ± 7.95). This group demonstrated greater stability compared to the low‐level group.

### 3.3 Univariate Analysis of the Trajectory of Professional Identity in Nursing Interns

As shown in Table 1, the two trajectory groups differed significantly in age, nursing as first‐choice major, attitude toward nursing, intention to pursue nursing career, and leadership experience (all *p* < 0.05).

### 3.4 Multivariate Analysis of the Trajectory of Professional Identity in Nursing Interns

All variables with *p* < 0.05 in univariate analyses were entered as candidate independent variables, including age, nursing as first‐choice major, attitude toward nursing major, intention to pursue nursing career, and leadership experience. Binary logistic regression was performed with nursing interns' professional identity trajectory group membership (low‐level group and high‐level group) as the binary dependent variable, which was coded as 1 for the low‐level group and 0 for the high‐level group. All above candidate variables were entered into the initial multivariate model, followed by backward elimination to remove predictors with *p* > 0.05 after adjustment. Age and intention to pursue a nursing career lost statistical significance in the adjusted model and were excluded from the final model. The regression results of the retained predictors showed that voluntarily choosing nursing as a university major (reference = no, OR = 0.195, *p* = 0.002), liking nursing (reference = dislike, OR = 0.260, *p* < 0.001), and having leadership experience (reference = no, OR = 0.446, *p* = 0.007) were all associated with lower odds of being classified into the low‐level group (all OR < 1). Based on the defined coding scheme for the dependent variable, these results suggested that interns with the above three characteristics were more likely to fall into the high‐level group. All final multivariate regression results are displayed in Table 3.

**TABLE 3:** Binary logistic regression of the trajectory of professional identity in nursing interns.

Characteristic	*β*	standard error	Wald *χ*2	OR(95% CI)	*P*
Nursing as first‐choice major (Ref = No)	−1.636	0.531	9.509	0.195 (0.069, 0.551)	0.002
Attitude toward nursing major (Ref = Dislike)					
Like	−1.346	0.310	18.857	0.260 (0.142, 0.478)	< 0.001
General	−0.421	0.886	0.226	0.656 (0.116, 3.725)	0.634
Leadership experience (class cadre/intern leader) (Ref = No)	−0.807	0.299	7.263	0.446 (0.248, 0.802)	0.007

*Note:* 1. Age and intention to pursue a nursing career showed statistical significance in univariate analysis (Table 1) but were excluded from the final multivariate logistic regression model due to severe multicollinearity (VIF > 10). All remaining variables retained in the regression model are listed above. 2. Dependent variable coding: 1 = low‐level group, 0 = high‐level group. OR < 1 indicates reduced odds of belonging to the low‐level group (higher odds of being in the high‐level group).

## 4 Discussion

### 4.1 The Current Status of Professional Identity Level in Nursing Interns

Our study showed that the level of professional identity of undergraduate nursing interns is at a moderate level, which is higher than that of Wu et al. (Wu et al. 2020). However, a clearer pattern emerged when examining the trajectory over time: scores increased from the early to the middle stage, then slightly decreased in the late stage, though remaining above the initial level. The initial low scores likely reflect the stress of role transition and limited clinical competence. This finding aligns with Najafi Kalyani et al. (Najafi Kalyani et al. 2019), who documented that early clinical exposure often triggers fear, confusion, and interpersonal conflicts, contributing to self‐doubt and diminished professional identity. The mid‐internship improvement may be attributed to growing clinical competence and environmental familiarity. As Fitzgerald et al. (Fitzgerald and Clukey 2021) observed, as interns gain proficiency and autonomy, they experience a greater sense of self‐efficacy and professional fulfilment. The slight decline observed in the later stage is consistent with research by Yi et al. (Yi et al. 2024) and Dong et al. (Dong et al. 2023), who attributed this phenomenon to accumulating burnout, compassion fatigue, and intensified job‐seeking pressure, which may undermine professional identity even when professional attitudes remain relatively stable. In the latter stages of their internship, interns have typically established their professional attitudes. However, they are susceptible to burnout, empathy fatigue, and employment pressures, which may lead to a slight decrease in their level of professional identity (Yi et al. 2024; Dong et al. 2023).

### 4.2 Two Trajectories of Professional Identity in Nursing Interns

Our LCGM analysis identified two distinct trajectories, confirming the existence of significant inter‐individual heterogeneity. The low‐level group exhibited a characteristic pattern of initial low scores, a mid‐internship rise, and a late‐stage decline, though scores remained above the initial level, suggesting susceptibility to fluctuation. This pattern may be explained by pre‐existing professional cognitions (Teresa‐Morales et al. 2021). Interns entering their placement with positive perceptions of nursing are likely to build professional confidence, whereas those with negative preconceptions may experience diminished enthusiasm and reinforce professional biases (Teresa‐Morales et al. 2021). Furthermore, this finding underscores the need for early identification of interns at risk and the development of tailored support strategies, particularly during the transition to the late internship phase. Consequently, it is imperative to promptly identify the low‐level group and formulate appropriate intervention strategies for this cohort.

### 4.3 Influencing Factors of the Professional Identity Trajectory in Nursing Interns

Our finding that voluntarily choosing nursing as a major and holding a positive attitude toward it were associated with higher professional identity is consistent with prior research (Zeng et al. 2022). A voluntary major choice and positive attitude likely reflect intrinsic motivation and initial professional commitment, which can sustain learning engagement and work enthusiasm throughout the internship, thereby reinforcing professional identity (Wei et al. 2021; Mao et al. 2021). This is corroborated by Wei et al. (Wei et al. 2021), who found that nursing interns who chose their major voluntarily reported significantly higher professional identity scores than those who followed parental wishes or institutional adjustments. Furthermore, personal interest in the major has been shown to enhance learning motivation and clinical problem‐solving ability, which in turn reinforces a sense of competence and value, further strengthening professional identity (Qtait 2026). Leadership experience also emerged as a significant predictor. This may be because students who take on such roles often demonstrate greater academic achievement, learning initiative, and responsibility. Research supports a positive link between professional identity and learning motivation (Jin et al. 2023). Additionally, leadership roles may foster a heightened sense of responsibility and recognition, which can further enhance professional commitment.

### 4.4 Implications for Nursing Education and Practice

The development of professional identity among nursing interns is a systematic process that depends on both external educational interventions and individual attributes, such as voluntary major choice, professional interest, and leadership experience. Role modelling is a core intervention for reshaping interns' occupational perceptions and consolidating professional identity. Mature implementation pathways include standardized university‐hospital collaborative orientation, narrative pedagogy with role modelling, and systematic professional image literacy training (Torres et al. 2022; Shen et al. 2026; Byram et al. 2022). Through experience sharing by senior nurses and nursing‐themed educational activities, such value‐oriented education can effectively counter negative social stereotypes, help interns develop positive professional perceptions, and strengthen their professional identification. Favourable individual traits—such as voluntary major choice, genuine passion for nursing, and leadership experience—can enhance interns' engagement with and internalization of educational interventions, thereby optimizing professional identity development. In the late internship period, targeted career planning courses and hospital‐based psychological counselling services can alleviate burnout and employment pressure, help interns clarify their long‐term career development paths (Fan et al. 2025), and provide essential support for stabilizing professional identity. For nursing interns with low professional identity—particularly those who did not voluntarily choose nursing, lack interest in the profession, or have no leadership experience—educators can implement targeted strategies based on existing evidence (1) Competency‐based clinical feedback: Tools such as mini‐CEX and DOPS provide specific, timely feedback on clinical skills. Mastery of practical skills can substantially enhance interns' professional self‐efficacy and facilitate stable professional identity formation (Xu et al. 2025); (2) Mentorship and peer support: Formal mentorship programs with qualified preceptors and counsellor‐facilitated peer support groups can provide one‐on‐one guidance and a safe space for experience sharing and emotional adjustment (Albaz et al. 2026); (3) Resilience training: Evidence‐based workshops on transition shock, compassion fatigue prevention, and psychological resilience—including mindfulness and cognitive reframing—can help interns cope with clinical stress (Qtait 2026); (4) Early career counselling: Proactive counselling can guide interns in exploring diverse nursing career paths that align with their personal interests, clarify the multidimensional development prospects of nursing, and ultimately consolidate professional commitment (Bikay et al. 2026).

## 5 Limitation

This study has several limitations that should be acknowledged when interpreting the results. First, the use of convenience sampling from only two tertiary hospitals in Anhui Province limits the generalisability of our findings. Regional variations in clinical training, workforce culture, and societal perceptions of nursing may yield different trajectory patterns, and our results may not be representative of interns in other settings (e.g., secondary hospitals or community health centres). Second, although our sample size met the minimum requirement, it remains modest. A larger, multi‐centre sample would enhance the robustness and external validity of the trajectory classes and predictor estimates. Third, our analysis was limited to demographic and experiential factors and did not incorporate subjective psychological constructs (e.g., self‐efficacy, career calling, resilience) or contextual variables (e.g., quality of supervision, hospital climate), which may also influence trajectory patterns. Fourth, relying exclusively on a self‐report questionnaire (PIQNS) may introduce response bias, such as social desirability. Future studies could incorporate qualitative interviews or supervisor evaluations to obtain a more comprehensive assessment. Future research should address these limitations by adopting multi‐centre sampling, integrating psychological and contextual predictors, and employing mixed‐methods designs to triangulate findings and deepen understanding of the mechanisms underlying professional identity development.

## 6 Conclusion

This study examined the dynamic evolution of professional identity among undergraduate nursing interns and identified two distinct trajectory classes: a high‐level, relatively stable group (49%) and a low‐level group with a notable late‐stage decline (51%). Predictors of membership in the high‐level group included voluntary major choice, a positive attitude toward nursing, and prior leadership experience. These findings underscore the need for collaboration between academic institutions and clinical settings to implement pre‐internship orientation and targeted, stage‐specific support—particularly for interns at risk of decline—to strengthen professional identity and reduce workforce attrition. Future research could expand to multi‐centre samples and explore psychological mediating factors to enhance result generalisability.

## Author Contributions

**Peng Shao:** conceptualization, methodology. **Hongrang Chen:** project administration, funding acquisition. **Biaoxin Zhang:** writing – review and editing, project administration, funding acquisition, resources. **Zhengju Chen:** conceptualization, methodology, software, data curation, formal analysis, writing – original draft.

## Funding

The Provincial Quality Engineering Project for Higher Education Institutions in Anhui Province (No 2024sx246). The Provincial Quality Engineering Project for Higher Education Institutions in Anhui Province (No 2024jyxm0745). Ideological and Political Project of the Innovation Center for Higher Education Institutions in Anhui Province (No AHSGSZX2025Z03).

## Ethics Statement

This study strictly adhered to the ethical principles outlined in the Declaration of Helsinki and was approved by the Ethics Committee of the First Affiliated Hospital of Anhui Medical University (Ethics Approval No: PJ2025‐04‐61) prior to initiating any data collection. All participants were undergraduate nursing interns who met the study’s inclusion criteria, and written informed consent was obtained from each individual before their enrolment.

## Conflicts of Interest

The authors declare no conflicts of interest.

## Data Availability

Research data are not shared.

## References

[nop270738-bib-0001] Albaz, N. , S. Alghumaiz , S. Nasser , and A. Althubaiti . 2026. “Experience and Perceived Requirements of a Peer Mentoring Program in Undergraduate Nursing Education: Insights From Faculty and Students.” BMC Nursing, 25, no. 1: 498. 10.1186/s12912-026-04822-6. Epub ahead of print. PMID: 42231251. 42231251 PMC13445696

[nop270738-bib-0002] Bikay, A. N. , O. P. Akpoveso , C. J. Nweke , E. C. Ekeoma , P. B. Tambe , and A. Kabiru . 2026. “Exploring Factors Influencing Medical Specialty Choice Among Undergraduates in The Gambia: A Situated Expectancy‐Value Theory Approach.” BMC Medical Education 26, no. 1: 883. 10.1186/s12909-026-09163-1. PMID: 41981561; PMCID: PMC13224476. 41981561 PMC13224476

[nop270738-bib-0003] Byram, J. N. , R. M. Frankel , J. H. Isaacson , and N. Mehta . 2022. “The Impact of COVID‐19 on Professional Identity.” Clinical Teacher 19, no. 3: 205–212. 10.1111/tct.13467. PMID: 35142075; PMCID: PMC9115480. 35142075 PMC9115480

[nop270738-bib-0004] Dong, C. , L. Xia , C. Zhao , et al. 2023. “Prospective Association Between Perceived Stress and Anxiety Among Nursing College Students: The Moderating Roles of Career Adaptability and Professional Commitment.” BMC Psychiatry 23, no. 1: 388. 10.1186/s12888-023-04887-6. PMID: 37264378; PMCID: PMC10234240. 37264378 PMC10234240

[nop270738-bib-0005] Fan, L. H. , G. H. Wang , J. M. Lei , C. Shi , and L. J. Yi . 2025. “The Role of Professional Identity, Psychological Resilience, and Coping Styles in Mitigating Compassion Fatigue Among Geriatric Services and Management Interns.” Geriatric Nursing 72: 103372. 10.1016/j.gerinurse.2025.05.011. PMID: 40383673. 40383673

[nop270738-bib-0006] Fitzgerald, A. , and L. Clukey . 2021. “Professional Identity in Graduating Nursing Students.” Journal of Nursing Education 60, no. 2: 74–80. 10.3928/01484834-20210120-04. PMID: 33528577. 33528577

[nop270738-bib-0007] Hao, Y. 2011. ‘Study of the model of self‐education in enhancing the level of professional identity and professional self‐efficacy in nurse students [Dissertation].’. Shanghai: The Second Military Medical University.

[nop270738-bib-0008] Jin, T. , Y. Zhou , and L. Zhang . 2023. “Job Stressors and Burnout Among Clinical Nurses: A Moderated Mediation Model of Need for Recovery and Career Calling.” BMC Nursing 22, no. 1: 388. 10.1186/s12912-023-01524-1. PMID: 37853383; PMCID: PMC10583433. 37853383 PMC10583433

[nop270738-bib-0009] Khattar, B. , A. Sharma , G. Tomer et al. 2025. “Knowledge, Awareness, and Attitude on Malocclusion in Nursing Students.” Journal of Pharmacy & Bioallied Sciences 17, no. Suppl 1: S326–S328. 10.4103/jpbs.jpbs_1929_24. PMID: 40510978; PMCID: PMC12156587. 40510978 PMC12156587

[nop270738-bib-0010] Mao, A. , S. E. Lu , Y. Lin , and M. He . 2021. “A Scoping Review on the Influencing Factors and Development Process of Professional Identity Among Nursing Students and Nurses.” Journal of Professional Nursing 37, no. 2: 391–398. 10.1016/j.profnurs.2020.04.018. PMID: 33867096. 33867096

[nop270738-bib-0011] Mukherjee, A. , A. Saravanan , A. Kamaraj , S. Thiyagarajan , A. Mohan , and A. Ramachandran . 2024. “A Correlational Study on Professional Identity and Self‐Efficacy Among Nursing Students.” Cureus 16, no. 8: e67508. 10.7759/cureus.67508. PMID: 37663091; PMCID: PMC10432710. 39310542 PMC11416135

[nop270738-bib-0012] Najafi Kalyani, M. , N. Jamshidi , Z. Molazem , C. Torabizadeh , and F. Sharif . 2019. “How Do Nursing Students Experience the Clinical Learning Environment and Respond to Their Experiences? A Qualitative Study.” BMJ Open 9, no. 7: e028052. 10.1136/bmjopen-2018-028052. PMID: 31350243; PMCID: PMC6661598. PMC6661598 31350243

[nop270738-bib-0013] Qtait, M. 2026. “Reframing Postgraduate Nursing Education: From Academic Attainment to Societal Impact: A Commentary.” SAGE Open Nursing 12: 23779608261457209. 10.1177/23779608261457209. PMID: 42232793; PMCID: PMC13223344. 42232793 PMC13223344

[nop270738-bib-0014] Rodríguez‐García, M. C. , L. Gutiérrez‐Puertas , G. Granados‐Gámez , G. Aguilera‐Manrique , and V. V. Márquez‐Hernández . 2021. “The Connection of the Clinical Learning Environment and Supervision of Nursing Students With Student Satisfaction and Future Intention to Work in Clinical Placement Hospitals.” Journal of Clinical Nursing 30, no. 7–8: 986–994. 10.1111/jocn.15716. PMID: 33487377. 33432645

[nop270738-bib-0015] Shen, X. , R. Bu , M. Geng , and Y. Chen . 2026. “A Mixed‐Methods Evaluation of a Narrative Medicine Course and Its Impact on Empathy Development Among Medical Students.” BMC Medical Education, 26, no. 1: 980. 10.1186/s12909-026-09584-y. Epub ahead of print. PMID: 42231272. 42231272 PMC13471609

[nop270738-bib-0016] Teresa‐Morales, C. , J. D. González‐Sanz , and M. Rodríguez‐Pérez . 2021. “Components of the Nursing Role as Perceived by First‐Year Nursing Students.” Nurse Education Today 102: 104906. 10.1016/j.nedt.2021.104906. PMID: 33892265. 33892265

[nop270738-bib-0017] Torres, D. A. , L. Jeske , S. J. Marzinski , R. Oleson , and M. L. Hook . 2022. “Best Fit Orientation: An Innovative Strategy to Onboard Newly Licensed Nurses.” Journal for Nurses in Professional Development 38, no. 6: 350–359. 10.1097/NND.0000000000000784. Epub 2021 Jul 30. PMID: 34334735. 34334735

[nop270738-bib-0018] Wei, L. Z. , S. S. Zhou , S. Hu , Z. Zhou , and J. Chen . 2021. “Influences of Nursing Students Career Planning, Internship Experience, and Other Factors on Professional Identity.” Nurse Education Today 99: 104781. 10.1016/j.nedt.2021.104781. PMID: 33527491. 33530029

[nop270738-bib-0019] Wu, C. , M. H. Palmer , and K. Sha . 2020. “Professional Identity and Its Influencing Factors of First‐Year Post‐Associate Degree Baccalaureate Nursing Students: A Cross‐Sectional Study.” Nurse Education Today 84: 104227. 10.1016/j.nedt.2019.104227. PMID: 31647333. 31683135

[nop270738-bib-0020] Xu, B. , X. Xing , G. Zhang , et al. 2025. “Effect of the Situation‐Background‐Assessment‐Recommendation Model Combined With the Mini‐CEX Assessment on the Clinical Communication, Clinical Thinking and Comprehensive Clinical Abilities of Nursing Interns.” BMC Medical Education 25, no. 1: 1596. 41233887 10.1186/s12909-025-08022-9 PMC12613504

[nop270738-bib-0021] Yi, L. , T. Shuai , J. Zhou , L. Cheng , M. F. Jiménez‐Herrera , and X. Tian . 2024. “Development and Validation of a Machine Learning‐Based Predictive Model for Compassion Fatigue in Chinese Nursing Interns: A Cross‐Sectional Study Utilizing Latent Profile Analysis.” BMC Medical Education 24, no. 1: 1495. 10.1186/s12909-024-06505-9. PMID: 39702113; PMCID: PMC11656937. 39702113 PMC11656937

[nop270738-bib-0022] Zeng, L. , Q. Chen , S. Fan , et al. 2022. “Factors Influencing the Professional Identity of Nursing Interns: A Cross‐Sectional Study.” BMC Nursing 21, no. 1: 200. 10.1186/s12912-022-00983-2. 35879704 PMC9310353

[nop270738-bib-0023] Zeng, X. , F. Yang , X. Xu , et al. 2023. “Employment Intention and Career Planning of Male Nursing Students in Different Levels of Colleges and Universities: A Qualitative Study.” Nursing Open 10, no. 12: 7659–7667. 37823429 10.1002/nop2.2006 PMC10643848

[nop270738-bib-0024] Zhao, C. , Y. Wang , X. Jia , et al. 2024. “Associations of Dietary Diversity Trajectories With Frailty Among Chinese Older Adults: A Latent Class Trajectory Analysis Based on a CLHLS Cohort.” Nutrients 16, no. 10: 1445. 38794683 10.3390/nu16101445 PMC11124478

[nop270738-bib-0025] Zhao, S. , Q. Liang , H. Tao , et al. 2024. “Transition Shock Among Nursing Interns and its Relationship With Patient Safety Attitudes, Professional Identity and Climate of Caring: A Cross‐Sectional Study.” BMC Nursing 23, no. 1: 64. 10.1186/s12912-024-01722-5. 38267964 PMC10807204

[nop270738-bib-0026] Zoccali, C. , and G. Tripepi . 2025. “Longitudinal Studies: Focus on Trajectory Analysis in Kidney Diseases.” Journal of Nephrology 38, no. 2: 435–444. 10.1007/s40620-024-01437-7. PMID: 38013889. 39644432

